# Trauma Coping Self-Efficacy Mediates Associations Between Adult Attachment and Posttraumatic Stress Symptoms

**DOI:** 10.3389/fpsyg.2022.799608

**Published:** 2022-03-07

**Authors:** Margaret Morison, Charles C. Benight

**Affiliations:** ^1^Department of Psychology, University of Colorado, Colorado Springs, Colorado Springs, CO, United States; ^2^Lyda Hill Institute of Human Resilience, University of Colorado, Colorado Springs, Colorado Springs, CO, United States

**Keywords:** attachment, social cognitive theory, coping self-efficacy, posttraumatic stress, trauma

## Abstract

Attachment orientations reflect individuals’ expectations for interpersonal relationships and influence emotion regulation strategies and coping. Previous research has documented that anxious and avoidant attachment orientations have deleterious effects on the trauma recovery process leaving these survivors vulnerable to posttraumatic stress disorder (PTSD) symptoms. However, avoidant attachment may be more complicated. Prior work has also found those high in avoidant attachment but also low in anxious attachment (i.e., dismissing) may not experience such vulnerabilities. Further, avoidant attachment individuals often report higher self-efficacy than their anxiously attached counterparts. The present study examined trauma coping self-efficacy (CSE-T) as a previously unexamined mechanism of action between adult attachment and PTSD symptoms. Structural equation modeling results showed that anxious attachment was associated with lower CSE-T and greater PTSD symptoms six weeks later. Further, a significant indirect effect of anxious attachment on PTSD symptoms through CSE-T was found. Contrary to hypotheses, avoidant attachment also exhibited an indirect effect on PTSD symptoms through CSE-T, such that avoidant attachment was associated with lower CSE-T, which in turn, was associated with greater PTSD symptoms. Also contrary to hypotheses, the interaction between anxious and avoidant attachment was not significantly associated with either CSE-T or PTSD symptoms. Results suggest that both anxious and avoidant attachment orientations contribute to poor self-regulation following trauma, as they undermine perceptions of trauma coping self-efficacy.

## Introduction

Early caregiving relationships influence how individuals self-regulate in the face of potential threats and remain influential into adulthood (Mikulincer and Shaver, 2016). Given the relationship between attachment orientations and the regulation of distress, researchers have examined the association between adult attachment and trauma adaptation. Researchers have suggested that the perceived (and often objective) threat faced by trauma survivors elicits the retrieval of pre-existing attachment representations that differ depending on previous experiences one has with close relationships (Mikulincer and Shaver, 2016). The current literature does not provide a clear understanding of how attachment styles influence both initial reactions to trauma as well as posttraumatic adaptation over time. In this paper we argue that perceptions of coping self-efficacy (CSE) in dealing with the initial trauma and the recovery process is a key mechanism by which posttraumatic stress disorder (PTSD) symptoms alleviate or worsen during recovery (Benight and Bandura, 2004). However, different levels of CSE perceptions across attachment orientations and the potential mediating role of CSE have yet to be examined. The present study attempts to fill this void.

Adult attachment theory provides a critical framework for understanding the role of interpersonal relationships with various attachment figures (e.g., partners, family) in the formation of working models of one’s self and others that influences subsequent proximity-seeking behaviors (Bowlby, 1982). A dominant model of adult attachment describes attachment orientations along two continuous attachment dimensions – anxious and avoidant attachment (Brennan et al., 1998; Fraley et al., 2015). Securely attached adults, who are low on both anxious and avoidant attachment, can maintain autonomy while also utilizing relational support when needed (Mikulincer and Shaver, 2016). Those who are anxiously attached are overwhelmed with concerns about being supported when in need, whereas avoidantly attached adults tend to distrust others and maintain distance in close relationships (Mikulincer et al., 2006). Anxious and avoidant attachment styles are often referred to as insecure attachment.

Bartholomew and Horowitz (1991) suggested a four-factor model of adult attachment that allows an additive combination of scores along the two insecure attachment orientations. Those high in anxious attachment but low in avoidant attachment are described as preoccupied, whereas those high in avoidant attachment and low in anxious attachment are called dismissing. An individual scoring highly on both anxious and avoidant dimensions are characterized as fearful. Consistent with the two-dimensional model of attachment (e.g., Brennan et al., 1998), individuals low in both anxious and avoidant attachment are securely attached. These attachment orientations manifest unique cognitive representations of relationships as well as behaviors related to support interactions.

Anxiously attached adults generally see the world as unsafe, supports as unreliable, and themselves as unable to cope with potential threats or unworthy of support (Mikulincer and Shaver, 2016). These individuals may utilize hyperactivating relational strategies (e.g., overdependence on close relationships, clinging or attempts to control behaviors of loved ones) (Mikulincer and Shaver, 2016).

Avoidantly attached individuals, in contrast, tend to distrust others and do not perceive seeking support as a viable option for managing distress. Driven by a desire to control or maximize distance from close relationship partners, avoidantly attached individuals attempt to evade intimacy or self-disclosure (i.e., referred to as deactivating strategies). Fraley and Shaver (1997) suggested that those who exhibit a prototypically dismissing (high avoidant, low anxious) attachment orientation are skilled at suppressing attachment-related distressing thoughts, as exhibited by decreases in physiological arousal following instructions to suppress the thought of abandonment by a partner. Overall, it is unclear as to whether the deactivating strategies employed by avoidantly attached individuals are effective methods for coping with stress.

In meta-analytic review by Woodhouse et al. (2015), adult attachment orientations separately were found to be significantly positively associated with PTSD symptoms across 46 cross-sectional and longitudinal studies. The estimated population effect size estimates were medium strength (secure $\hat{\rho }$ = −0.27, anxious $\hat{\rho }$ = 0.26, and avoidant$\hat{\rho }$ = 0.24). Among studies within the meta-analytic review examining the four-factor model of attachment, dismissing attachment orientation (high avoidant, low anxious) was not significantly associated with PTSD symptoms ($\hat{\rho }$ = 0.16), whereas preoccupied (low avoidant, high anxious) and fearful (high avoidant, high anxious) attachment orientations were (preoccupied $\hat{\rho }$ = 0.31, fearful $\hat{\rho }$ = 0.44). Unfortunately, the authors did not test a moderating effect for study design (cross-sectional vs. longitudinal). These four-factor findings suggest that the relationship between an avoidant attachment orientation and PTSD symptoms may also depend on the level of anxious attachment.

Longitudinal studies have similarly demonstrated the influence of attachment orientations on PTSD symptoms. Within the types of insecure attachment, longitudinal results on the association between insecure attachment styles and PTSD symptoms were largely consistent, such that anxious and avoidant attachment were positively associated with PTSD symptoms (Fraley et al., 2006; Solomon et al., 2008; Franz et al., 2014; Shallcross et al., 2014; Murphy et al., 2016; see also Besser and Neria, 2010). However, among the two longitudinal studies to examine the interaction between anxious and avoidant attachment (Fraley and Bonanno, 2004; Fraley et al., 2006), findings regarding the relationship between a dismissing attachment orientation (high avoidant, low anxious) and PTSD symptoms have been mixed. More research on this interaction between avoidant and anxious attachment orientations and its influence on PTSD symptoms, as well as potential mechanisms of action, is needed.

An important potential mechanism of action are individuals’ perceptions of their capabilities in the face of external and internal threat. The perceived ability to cope (i.e., perceived CSE) in the face of environmental demands is a crucial determinant of recovery (Benight and Bandura, 2004).

Indeed, the predictive utility of CSE was demonstrated by two meta-analytic reviews (Luszczynska et al., 2009; Gallagher et al., 2020). Luszczynska et al. (2009) found that, among longitudinal studies, CSE predicted PTSD symptoms and general distress (distress, depression, and anxiety) up to 8 months post-event with medium to large effect sizes (general distress *r* = −0.50, PTSD *r* = −0.62). The effect sizes were also significant but more varied in cross-sectional studies. Greater CSE predicted lower PTSD symptom severity (*r* = −0.36), frequency (*r* = −0.77), and lower general distress (*r* = −0.50). More recently, Gallagher et al. (2020) similarly found a medium to large effect of CSE on PTSD symptom severity among cross-sectional (weighted *r* = −0.49) and longitudinal (weighted *r* = −0.52) studies. Collectively, CSE demonstrates a relatively stronger effect compared to other psychosocial predictors of PTSD, such as perceived social support (*r* = −0.28; Ozer et al., 2003) and emotion dysregulation (*r* = 0.53; Seligowski et al., 2015).

As outlined in Social Cognitive Theory (SCT; Bandura, 1997), the belief that one can exercise control over the challenges of trauma recovery will influence how one perceives potential threats, the choices they make, and how resilient they are to stressors (Benight and Bandura, 2004). Those with higher CSE are more likely to utilize adaptive coping strategies and maintain an active role in shaping their environment to facilitate recovery. Conversely, lower CSE leads to being more sensitive to possible environmental threats, and in turn, fears about managing these threats. The ultimate effect of lower CSE is impaired functioning and greater PTSD symptom severity.

Previous research on attachment has alluded to potential relationships between attachment orientations and CSE. For example, Mikulincer and Florian (1995) found anxious attachment to be associated with poor self-efficacy perceptions regarding the ability to cope with military training. Sheinbaum et al. (2015) also reported that anxiously attached individuals reported more negative appraisals about themselves and their capacity to cope with daily living. Experimental research also has demonstrated that anxiously attached adults hold less positive self-appraisals when under distress (Mikulincer, 1998; Vrtička et al., 2012). When managing environmental challenges, anxiously attached adults appear to evaluate themselves as less capable and, in turn, have difficulties self-regulating distress (Mikulincer and Shaver, 2016). Through the self-regulatory process involved with managing posttraumatic demands, it is reasonable to suggest that anxiously attached individuals would have lower CSE as they struggle with relational support (Benight and Bandura, 2004).

Avoidantly attached individuals may have a very different relationship with CSE. Mikulincer and colleagues proposed that avoidantly attached people may possess greater self-efficacy regarding their ability to maintain distance through coping mastery they have achieved without depending upon others (Mikulincer and Florian, 1998; Mikulincer et al., 1999). If one has internal working models of others as typically unavailable and unhelpful when one is distressed, self-reliance may provide one with more positive appraisals regarding the ability to cope on one’s own (“I don’t need anyone!”). Interestingly, avoidantly attached adults have demonstrated positive self-appraisals (Mikulincer and Florian, 1995; Mikulincer, 1998), but also negative self-appraisals (Sheinbaum et al., 2015). One possible answer to these disparate findings may be the level of anxious attachment that coexists with avoidant style (e.g., Fraley and Shaver, 1997).

Although other mediators in the attachment-PTSD relationship have been identified, such as perceived social support (Besser and Neria, 2010, 2012), coping strategies (Mikulincer et al., 1993) and emotion regulation (Benoit et al., 2010), one could argue that CSE plays a key role in explaining how these constructs exert an influence on individuals’ responses to stress. Perceived social support boosts CSE perceptions (enabling hypothesis), thus reducing distress (Schwarzer and Knoll, 2007; Smith et al., 2013, 2017). However, the influence of attachment orientations on CSE perceptions and PTSD symptoms in trauma survivors has yet to be examined. This study attempts to fill this void.

**FIGURE 1:**
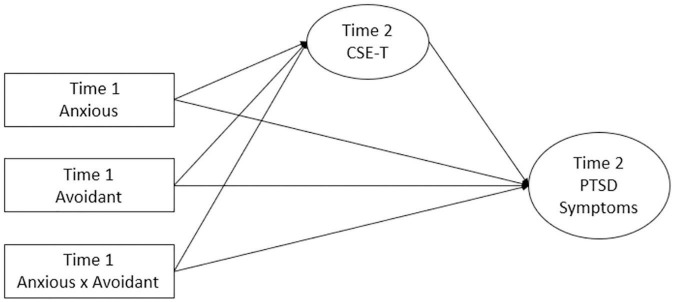
Conceptual Model Predicting PTSD Symptoms at Six Weeks. CSE-T = Trauma Coping Self-Efficacy; PTSD = Posttraumatic stress disorder.

Thus, we hypothesized that trauma-related CSE (CSE-T) perceptions (measured at follow-up) would mediate the relationship between baseline anxious attachment and PTSD symptoms six weeks later (see Figure 1). For anxiously attached adults who experience negative self-evaluations (Mikulincer et al., 2006), persistent symptoms would result in greater decreases in CSE-T over time, given that the attempts at coping thus far have not alleviated symptoms. Low CSE-T would, in turn, serve to worsen PTSD symptoms (Benight and Bandura, 2004; Luszczynska et al., 2009; Bosmans and van der Velden, 2017). For avoidant attachment, we hypothesized that there would be a significant indirect effect on PTSD symptoms through CSE-T, such that avoidantly attached adults would experience a greater sense of CSE-T, which in turn would lessen PTSD symptoms. To examine potential relationships between with CSE-T and PTSD for those high in avoidant attachment and low in anxious attachment (e.g., dismissive attachment), an interaction term was used. A fearful attachment (high anxious, high avoidant), characterized by both a fear of rejection and avoidance of others (Bartholomew and Horowitz, 1991), would lack the benefits of a boosted self-efficacy when faced with distress, thus leading to the observed relationship between avoidant attachment and PTSD elsewhere (Fraley et al., 2006; Solomon et al., 2008; Franz et al., 2014; Murphy et al., 2016). Thus, we hypothesized that survivors whose attachment orientation were more prototypically dismissing would experience greater CSE-T and less PTSD symptoms, whereas those more fearful would experience low CSE-T and high PTSD symptoms. Given the importance of timing in the measurement of PTSD symptoms (e.g., Galatzer-Levy et al., 2018), time since the traumatic event was controlled for by recruiting individuals who had experienced a traumatic event in the last year and incorporating the time since trauma as a covariate in the present analyses.

## Materials and Methods

### Participants

The inclusion criteria for the present study consisted of (a) having experienced at least one traumatic event based on the DSM-5 (American Psychiatric Association [APA], 2013) definition in the last year, (b) being at least 18 years old, and (c) fluency in English. Participants (N_T1_ = 380, N_T2_ = 227) were primarily female (67.3%), White (72.5%), and married (38%; see Table 1). The present sample was well-educated, with the majority holding a college degree. Trauma exposure as measured by the Life Events Checklist (LEC; Weathers et al., 2013) revealed the most common traumatic events (either experienced, witnessed, learned of, or as part of job) were transportation accidents (70.7%), followed by life-threatening illness or injury (62.8%), other (62%), natural disasters (59.2%), physical assaults (57.3%), unwanted sexual experiences (55.8%). The least common traumatic events were causing harm or death to someone else (15.4%) and captivity (13.9%). A small percentage of participants reported receiving a COVID-19 diagnosis at any time during the pandemic (N_T1_ = 15, N_T2_ = 9). Six individuals reported receiving a COVID-19 diagnosis at T2 that was not reported at T1, which may indicate they became ill during the study. Time since the most currently distressing traumatic event ranged from 0 to 365 days, with the average amount of time passed being 197.73 days (*SD* = 107.48). Three participants (two healthcare workers, one exposed to domestic violence) were experiencing ongoing trauma exposure and were thus coded as “0” for the number of days since the traumatic event.

**TABLE 1:** Descriptives for demographic variables (*N* = 380).

Measures	*n*	%
**Gender**		
Male	116	30.4
Female	257	67.3
Non-binary	7	1.8
Other	2	0.5
**Ethnicity**		
White/Caucasian	277	72.5
Black/African American	30	7.9
Hispanic/Latinx	23	6.0
Indigenous American/Native American/Alaskan Native	3	0.8
Asian American/Pacific Islander	32	8.4
Multi-racial	15	3.9
Other	2	0.5
**Annual Household Income**		
Less than $15,000	49	12.9
15,000 to $35,000	80	21.0
35,000 to $50,000	84	22.0
50,000 to $75,000	70	18.4
75,000 to $100,000	50	13.1
100,000 or greater	48	12.6
**Education**		
High school	40	10.5
Some college	88	23.0
Associate’s degree	41	10.7
Bachelor’s degree	148	38.7
Master’s degree	55	14.4
Doctoral or professional degree	10	2.6
**Relationship status**		
Single (never married)	125	32.7
Committed partnership	86	22.5
Married	145	38.0
Separated	6	1.6
Divorced	15	3.9
Widowed	4	1.0
Other	1	0.3

### Procedure

**FIGURE 2:**
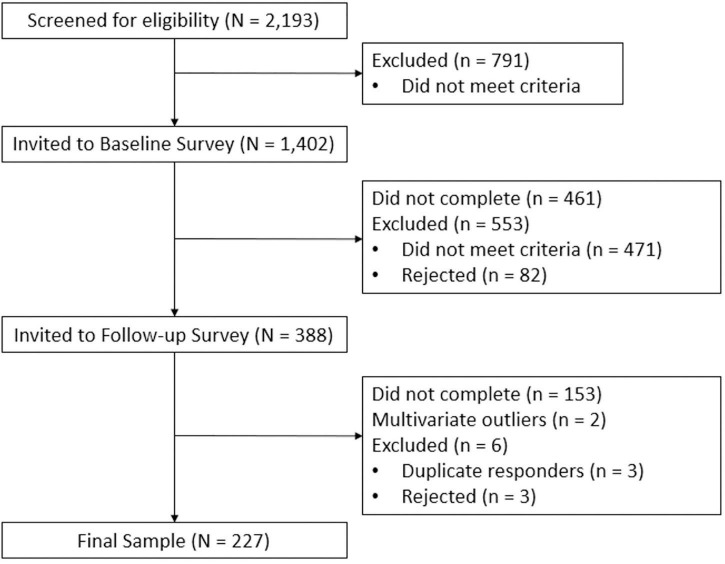
Participant Flow Chart.

Community participants were recruited through Amazon’s Mechanical Turk (MTurk), with data collection taking place between June and November 2020 (Morison, 2021). A longitudinal correlational design was utilized to test study hypotheses including a baseline survey and a six-week follow-up assessment. The surveys were created through the Cloud Research website, which offers a user-friendly interface for creating longitudinal Mturk surveys (Litman et al., 2017). Based on sample size recommendations by Weston and Gore (2006), we aimed to collect a minimum of 200 participants. In order to reach this minimum sample size, a substantially larger number of potential participants were screened for inclusion (see Figure 2). Participants were compensated for each survey, providing $0.02 for the screening survey, $1.00 for the baseline survey, and $2.00 for the follow-up survey.

Prior to informed consent and inclusion in the study, potential participants completed a brief screener survey with four yes or no questions to assess eligibility criteria. Those who self-identified as having met the inclusion criteria were invited to complete the baseline survey. Eligible participants completed self-report surveys on attachment, CSE, and PTSD symptoms at both baseline (T1) and a follow-up survey six weeks later (T2). As outlined in Figure 2, about one third of individuals who completed the baseline survey did not meet the trauma criteria or did not experience a traumatic event in the timeframe specified. The DSM-5 definition of a traumatic event specifies that the event must involve exposure to “…actual or threatened death, serious injury, or sexual violence, in one (or more) of the following ways: (1) Directly experiencing the traumatic event(s), (2) Witnessing, in person, the event(s) as it occurred to others, (3) Learning that the traumatic event(s) occurred to a close family member or close friend. In the case of actual or threatened death, the event(s) must have been violent or accidental, (4) Experiencing repeated or extreme exposure to aversive details of the traumatic event(s)” (American Psychiatric Association [APA], 2013, p 271). Of the 471 potential participants who did not meet inclusion criteria, 341 of these reported an event that was inconsistent with the DSM-5 definition of a traumatic event. These participants were not invited to complete the follow-up survey and were not included in analyses.

As careless responding and automated bots are of concern in Mturk studies, several checks were utilized to ensure the quality of the data collected. Duplicate IP addresses were blocked from completing any survey on multiple occasions through the Cloud Research service (Litman et al., 2017). However, three duplicates with matching Mturk user IDs were identified in the data cleaning process and responses were discarded. Open-ended questions regarding trauma exposure were included to (1) assess the most distressing traumatic event meets DSM-5 criteria and falls within the last year, and (2) provide confirmation that the survey was being completed by a person and not a bot. One common example of a rejected response to the open-ended trauma exposure question was providing a copy-pasted definition of PTSD. Blank responses to open-ended questions were not included in the present analyses as inclusion criteria could not be verified.

### Measures

#### Demographics

Participants reported on various demographic information, including age, gender, ethnicity, highest level of education, and annual household income. Participants were also asked to indicate whether they had received a diagnosis of COVID-19 due to the ongoing SARS-CoV-2 pandemic and the potential contribution to missing data.

#### Trauma Exposure

Exposure to a traumatic event in the last year was assessed using the extended version of the LEC (Weathers et al., 2013). The LEC lists 17 different traumatic events, from which participants are asked to indicate if they had experienced it themselves, witnessed the event, heard about the event occurring to a close friend or family member, was experienced through the course of a job (e.g., first responder), or was not experienced. The LEC has exhibited acceptable temporal stability and convergent validity with other established measures of trauma history, such as the Traumatic Life Events Questionnaire (Gray et al., 2004). The extended LEC also included open-ended questions regarding the worst event in the last year and how much time had elapsed since the traumatic event. These open-ended questions were carefully reviewed by the first author to ensure the event met DSM-5 criteria and the event fell within the last year. Corresponding to the DSM-5 definition of a traumatic event, participants who indicate any degree of trauma exposure were considered eligible to participate. Participants who described an event not meeting criteria, such as job loss or death of a loved one by natural causes, were not included in the present analyses.

#### Adult Attachment

Anxious and avoidant attachment was assessed using the Experiences in Close Relationships – Relationship Structures scale (ECR-RS; Fraley et al., 2011a). Participants were instructed to respond to the same nine statements regarding how they feel toward different attachment figures, including attachment with his or her romantic partner, close friend, mother figure, and father figure. For each attachment figure, the Anxious attachment subscale items emphasize concerns over abandonment (e.g., “I need a lot of reassurance that close relationship partners really care about me”), while the Avoidant attachment subscale items emphasize a desire to distance from close others (e.g., “I prefer not to be too close to others”). Items are rated from 1 (*Disagree strongly*) to 7 (*Agree strongly*) and summed on each subscale to create total scores for Anxious and Avoidant attachment. Mean composites, using averaged anxious or avoidant scores for each attachment figure, have exhibited good reliability (Anxious α = 0.80, Avoidant α = 0.88; Fraley et al., 2011a). Previous work by Fraley et al. (2011b) demonstrated that attachment orientation to each attachment figure (e.g., partner, friend, mother, and father) may be used as indicators for an overall latent construct representing one’s “global” or “prototypical” attachment orientation. This approach to estimating global attachment may result in less ambiguity as to whom the participant should be bearing in mind when responding to the self-report statements. Global scores for attachment anxiety and attachment avoidance in the present sample demonstrated good internal consistency (α = 0.91 and 0.90, respectively).

#### Posttraumatic Stress Disorder Symptoms

The PTSD Checklist for DSM-5 (PCL-5; Weathers et al., 2013) was used to measure PTSD symptoms. Items on the PCL-5 correspond to PTSD symptom criteria from the DSM-5, including intrusive symptoms, avoidance, negative alterations in cognition or mood, and arousal symptoms (American Psychiatric Association [APA], 2013). Participants were instructed to respond to the 20-item questionnaire regarding how bothered he or she was by symptoms in the past month concerning his or her experience in the past year (Weathers et al., 2013). Item responses range from 0 (*Not at all*) to 4 (*Extremely*). Items were summed to create a total PTSD symptom severity score. The PCL-5 has demonstrated good internal consistency (α = 0.95) and test-retest reliability (*r* = 0.82) over one week (Belvins et al., 2015). The PCL-5 total score demonstrates good internal consistency in the present sample, α = 0.96. Average T2 PTSD symptom severity exceeded the cut-off value of 33.0 suggesting probable PTSD diagnosis recommended by the National Center for PTSD (*M* = 47.93, *SD* = 19.74), suggesting that the present sample was highly distressed.

#### Trauma Coping Self-Efficacy

Trauma survivors’ CSE was measured using the Trauma Coping Self-Efficacy (CSE-T) self-report scale (Benight et al., 2015). The CSE-T is a 9-item, context-specific measure of survivors’ perceptions of his or her coping capabilities surrounding trauma symptoms (e.g., “Control thoughts of the traumatic experience happening again”) and other posttraumatic demands (e.g., “Get my life back to normal”). Participants were instructed to indicate the extent to which they feel capable of coping with these various posttraumatic demands. Items are rated on a 7-point scale ranging from 1 (*not at all capable*) to 7 (*totally capable*). The CSE-T has demonstrated good internal consistency (α = 0.87 to.91), discriminant validity, and test-retest reliability (*r* = 0.57 to.81). In the present sample, total CSE-T score demonstrated good internal consistency, α = 0.92.

### Data Analysis

Adult attachment variables were created by averaging avoidant and anxious scores across the different relational contexts (i.e., friend, mother, father, and partner; Fraley et al., 2011a). Additionally, an interaction term was formed using the product of mean-centered global avoidant attachment and global anxious attachment (Aiken and West, 1991). Latent variables were formed for CSE and PTSD symptoms, allowing for the removal of measurement error (Crano et al., 2015). Further, parceling was utilized to form latent variables, thereby minimizing the number of paths to be estimated in the measurement model in favor of parsimony (Matsunaga, 2008). To employ parceling for PTSD, symptom cluster mean composites were used as indicators for the PTSD symptom latent variable. Finally, the nine-item CSE-T was randomly parceled to form three mean composites with three items each. Details outlining the parcel items, internal consistency, means, and standard deviations of observed model variables are included in Table 2.

**TABLE 2:** Descriptives for Observed Variables for T1 (*N* = 380) and T2 (*N* = 227).

Scale	# of Items	α T1	α T2	*M*	*SD*
**ECR-RS**					
Attachment Anxiety	12	0.91	–	0.00	1.43
Attachment Avoidance	24	0.90	–	0.00	0.98
**CSE-T**					
Parcel 1 (Items 1, 6, 9)	3	–	0.76	4.61	1.40
Parcel 2 (Items 3, 5, 8)	3	–	0.82	4.44	1.44
Parcel 3 (Items 2, 4, 7)	3	–	0.75	4.93	1.32
**PCL-5**					
Cluster B	5	–	0.91	2.33	1.04
Cluster C	2	–	0.87	2.74	1.21
Cluster D	7	–	0.91	2.43	1.08
Cluster E	6	–	0.87	2.31	1.02

*ECR-RS = Experiences in Close Relationships Scale – Relationship Structures; CSE-T = Trauma Coping Self-Efficacy Scale; PCL-5 = The PTSD Checklist for the DSM-5.*

#### Model Estimation and Fit

A partially latent structural equation model (SEM) was estimated using Mplus software version 8.5 (Los Angeles, CA; Muthén and Muthén, 2017). A two-step process was utilized for estimating the proposed model (e.g., Mueller and Hancock, 2007). Prior to estimating the proposed structural model, the measurement portion of the model was estimated in order to address any model misspecification concerns. Several indices of model fit were used to determine how well the model structure fit the underlying data. The model χ^2^ was examined, which assesses the discrepancy between the hypothesized model and underlying covariate structure of the data. Model χ^2^ uses significance testing, wherein a significant χ^2^ indicates the model is significantly different from the underlying data. However, as a p-value test, model χ^2^ is sensitive to sample size (Kline, 2016). Thus, the adjusted model χ^2^/df was also examined, using a cutoff value of less than 3.00 (Kline, 1998). As indicators of goodness-of-fit, the Comparative Fit Index (CFI; Bentler, 1990) and Tucker-Lewis Index (TLI; Tucker and Lewis, 1973) were also examined. These indices compare the proposed model to the independence model (i.e., model with no estimated parameters). Values of CFI and TLI over 0.95 represent a good fitting model (Hu and Bentler, 1999). The Root Mean Square Error of Approximation (RMSEA; MacCallum et al., 1996) and Standardized Root Mean Square Residual (SRMR; Jöreskog and Sörbom, 1981) were used as “badness-of-fit” indices, which compare the proposed model to a saturated model (i.e., all possible parameters estimated). RMSEA and the accompanying 90% confidence interval utilize a cutoff of less than 0.08 to identify a good fit, and between 0.08 and 0.10 indicate a mediocre fit (MacCallum et al., 1996). Similarly, SRMR uses a cutoff of 0.08 to indicate a good fitting model (Hu and Bentler, 1999).

## Results

### Preliminary Analyses

#### Missing Data and Attrition

Item-level missing data and missing data due to dropout from the study (i.e., attrition) were examined to establish the degree of missingness and potential mechanisms of missingness. There was no item-level missing data for T1 or T2 on any model variables. However, there was 40% attrition. To identify the proper procedure for handling this degree of missing data due to attrition, we first sought to identify if the data was Missing Completely At Random (MCAR), Missing At Random (MAR), or Not Missing At Random (NMAR; Rubin, 1976; Little and Rubin, 2002). There were no significant differences between completers and dropouts on gender, ethnicity, annual household income, T1 COVID-19 diagnosis, time since trauma, T1 attachment anxiety global score, T1 attachment avoidance global score, T1 CSE-T total score, or T1 PTSD symptom total score. However, those who completed both T1 and T2 were significantly older (*M* = 34.62, *SD* = 10.36) than those who dropped out (*M* = 31.20, *SD* = 11.36), *t*(380) = −2.98, *p* < 0.01, Cohen’s *d* = −0.31. Given this significant group difference, a MCAR mechanism of missing data was not supported. Although there is no test to differentiate between MAR and NMAR, age was included as an auxiliary variable in the SEM in order to satisfy the assumptions of MAR (Enders, 2010). Auxiliary variables, including variables that are correlates of missingness, are often utilized to enhance the missing data procedure by reducing bias (Collins et al., 2001).

Full information maximum likelihood (FIML) was used to estimate missing data using Mplus version 8.5 (Muthén and Muthén, 2017). This method does not impute missing data points, instead it estimates model parameters and standard errors using all observed variables (Enders, 2001). Maximum likelihood (ML) methods demonstrate less biased performance as compared to listwise or pairwise deletion for MCAR or MAR data (Enders, 2001, 2010; Schafer and Graham, 2002), even under conditions of high rates of missing data (Enders, 2010, p. 95; Schafer and Graham, 2002). Further, FIML has been found to produce less biased parameter estimates and fit indices in SEM (Feng et al., 2012). Importantly, the inclusion of auxiliary variables (e.g., age) within a FIML estimation procedure make the MAR assumption more likely, thus yielding less biased estimates.

#### Normality and Outliers

Normality of observed variables was examined in SPSS 27. Skewness values for anxious attachment, avoidant attachment, CSE-T parcels, and PCL-5 parcels indicate these variables were relatively normally distributed, falling between −0.74 and 0.78. Kurtosis values fell between −1.01 and 0.22, providing another indication of relative normality. Mahalanobis distances was used to identify multivariate outliers. Two participants were identified as multivariate outliers at *p* < 0.001 and were subsequently removed.

#### Measurement Model

**FIGURE 3:**
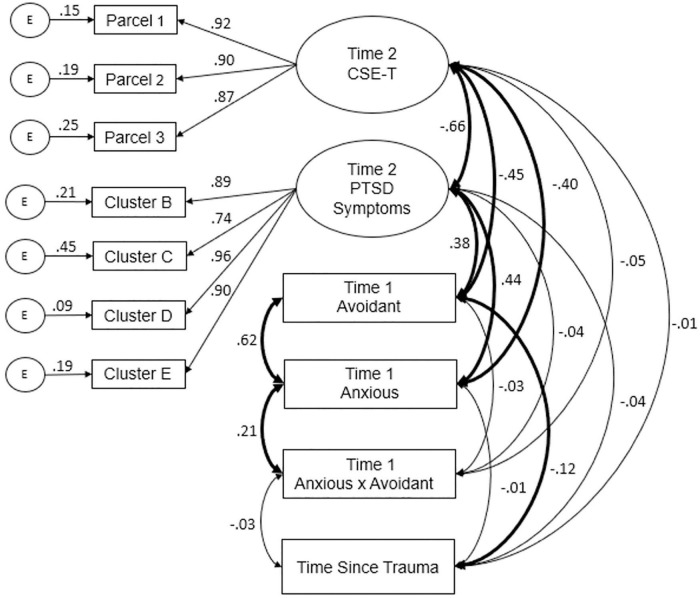
Measurement Model. Standardized factor loadings and covariances are presented. Bolded pathways are significant at *p* < 0.05.

As the first step of the estimation process, the measurement model was examined by covarying latent and non-latent variables (see Figure 3). Model fit indices suggested the model fit was acceptable, χ^2^(33) = 77.08, *p* < 0.001, χ^2^/df = 2.34, CFI = 0.98, TLI = 0.96, SRMR = 0.03, RMSEA = 0.06, 90% CI [0.04, 0.08]. Modification indices consisted of correlated error terms between the PTSD latent variable indicators and between indicators of the PTSD and CSE-T latent indicators. Incorporating error terms in this fashion is undesirable, as the model may simply become unique toward the specific sample used resulting in low replicability (Ho, 2006). Thus, these changes were not incorporated. Although the model exhibited a significant model χ^2^, the acceptable χ^2^/df value and presence of good fit amongst the other indices led to retainment of the measurement model.

Factor loadings for the CSE-T and PTSD latent variables were high, ranging from 0.87 to 0.92 for CSE-T and 0.74 to 0.96 for PTSD. Attachment anxiety and attachment avoidance were significantly positively correlated (*r* = 0.62), however, only attachment anxiety was significantly correlated with the interaction term (*r* = 0.21). Attachment anxiety was significantly correlated with CSE-T (*r* = −0.40) and PTSD (*r* = 0.44). Similarly, attachment avoidance was significantly correlated with CSE-T (*r* = −0.45) and PTSD (*r* = 0.38). Consistent with prior work, CSE-T exhibited a moderately strong, negative correlation with PTSD (*r* = −0.66). Interestingly, time since trauma was significantly negatively correlated with attachment avoidance (*r* = −0.12).

#### Structural Model

**FIGURE 4:**
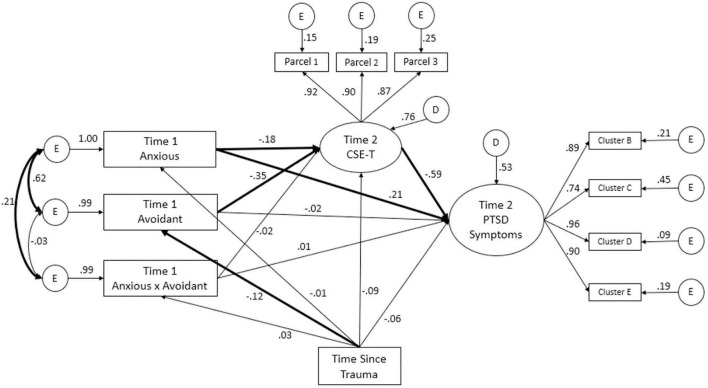
Structural Model. Standardized factor loadings and covariances are presented. Bolded pathways are significant at *p* < 0.05.

Direct and indirect effects of the structural model were estimated using 5,000 bootstraps (see Figure 4). Similarly to the measurement model, model fit indices suggested the model fit the data well, χ^2^(33) = 77.08, *p* < 0.001, χ^2^/df = 2.34, CFI = 0.98, TLI = 0.96, SRMR = 0.03, RMSEA = 0.06, 90% CI [0.04, 0.08]. Anxious attachment exhibited significant direct effects with both CSE-T (β = −0.18) and PTSD (β = 0.21) in expected directions. Avoidant attachment was not significantly directly associated with PTSD symptoms (β = −0.02). Further, avoidant attachment was significantly associated with CSE-T (β = −0.35). Finally, the interaction term between anxious and avoidant attachment was not significantly associated with either CSE-T or PTSD symptoms (β = −0.02 and 0.01, respectively). Thus, the hypotheses regarding avoidant attachment and the interaction term were not supported.

A significant indirect effect of anxious attachment on PTSD symptoms via CSE-T was found, β = 0.11, 95% CI [0.01, 0.22]. Consistent with hypotheses, anxious attachment was negatively associated with CSE-T, which in turn was negatively associated with PTSD symptoms. Similarly, a significant indirect effect of avoidant attachment on PTSD symptoms via CSE-T was found, β = 0.21, 95% CI [0.09, 0.33]. Specifically, greater avoidant attachment was negatively associated with later CSE-T, which in turn, was negatively associated with PTSD symptoms. No significant indirect effect of the interaction term on PTSD symptoms was found, β = 0.01, 95% CI [-0.08, 0.09].

## Discussion

The present study sought to integrate attachment theory (Bowlby, 1982) and SCT (Bandura, 1997) to better understand how adult attachment styles impact self-appraisals and self-regulation during the recovery process in a national sample of trauma survivors. CSE-T was a significant mediator between anxious attachment and later PTSD symptoms. In contrast to hypotheses, CSE-T also played a mediating role between avoidant attachment and PTSD symptoms. Finally, it was hypothesized that the interaction between anxious and avoidant attachment would be significantly associated with CSE-T and PTSD symptoms. Specifically, it was expected that those high in avoidant attachment but low in anxious attachment (e.g., dismissing) would have greater CSE-T and lower PTSD symptoms. However, results did not support the importance of the interaction between anxious and avoidant attachment with either CSE-T or PTSD symptoms. Results have important implications for the role of attachment in trauma recovery.

Anxious attachment was associated with lower levels of CSE-T. Consistent with the function of CSE in the process of self-regulation outlined by SCT (Bandura, 1997; Benight and Bandura, 2004), a significant indirect effect was also observed as predicted. Indeed, results suggest that CSE-T is an important self-regulation mechanism by which anxious attachment relates to subsequent PTSD symptoms. Findings here are consistent with prior work finding negative associations between anxious attachment and constructs closely related to CSE-T, such as perceived control over emotional responses (Vrtička et al., 2012), military training-related CSE (Mikulincer and Florian, 1995), and CSE in daily life (Sheinbaum et al., 2015). Though comparable to these related constructs, CSE-T represents a set of context specific self-appraisals more proximal to goals of coping with demands of trauma recovery.

The influence of anxious attachment on self-regulation plays an important role in adaptation from traumatic events. Anxious attachment is characterized by both a distorted fear of being abandoned in close relationships and negative appraisals of the self (Bowlby, 1982; Mikulincer and Shaver, 2016). The self-derogation typical of adults high in anxious attachment feeds into hyperactivating strategies as the individual attempts to regain a sense of security. However, such strategies appear ineffective as a method of self-regulation, as distress and appraisals of threat are high in those highly anxiously attached (Mikulincer et al., 1993; Mikulincer and Florian, 1995; Sheinbaum et al., 2015; Silva et al., 2015; Taylor et al., 2017). CSE-T may act as an explanatory mechanism through which these hyperactivating strategies exacerbate distress among trauma survivors high in anxious attachment.

From an SCT perspective, as individuals attempt to cope with posttraumatic recovery demands, those who struggle to maintain a sense of competence (i.e., low CSE-T) will struggle with effective coping and increasing levels of distress (Benight and Bandura, 2004). Indeed, as CSE-T decreases, there is a greater perception of potential threats over which one feels incapable of exerting control. CSE-T fills an important theoretical gap in explaining the process by which anxious attachment internal working models maintain distress. Whereas individuals high in anxious attachment continue to seek out supports (Mikulincer et al., 1993; Mikulincer and Florian, 1995; Vogel and Wei, 2005), they tend to perceive social support as less available due to the negative internal working models of anxious attachment (Besser and Neria, 2010, 2012). Thus, social supports fail to provide relational boosts to support individual coping capacity (e.g., Smith et al., 2013, 2017) driving down CSE-T appraisals and thereby exacerbating distress. The hyperactivating strategies exhibited by those high in anxious attachment appear to reflect this continuous unmet need for felt security, with CSE-T acting as a mechanism to partially explain such strategies’ ineffectiveness. Future work perhaps utilizing ecological momentary assessments (i.e., real-time, *in vivo* measurements typically obtained via Smartphone or App) that target relational dynamics may provide greater clarity concerning how anxiously attached individuals experience social support efforts and their own self-evaluative process.

Contrary to hypotheses, avoidant attachment was significantly associated with lower CSE-T instead of higher efficacy beliefs. Similar to anxious attachment, CSE-T acted as a mechanism by which avoidant attachment and PTSD symptoms were longitudinally associated. The role of avoidant attachment in coping with stress has been conceptualized as both potentially adaptive and a potential risk factor for greater distress. Indeed, it has been argued that the defensive responses exhibited by avoidantly attached individuals, including the suppression of attachment-related concerns (Fraley and Shaver, 1997) and positive self-appraisals (Mikulincer and Shaver, 2016) may be effective in minimizing distress. In contrast, others find that both forms of insecure attachment may make one vulnerable to difficulties adapting from trauma exposure (Fraley et al., 2006; Franz et al., 2014; Shallcross et al., 2014). The results of the present study lend support to the latter conceptualization.

The relationship between avoidant attachment and self-appraisals has largely indicated that avoidant attachment is associated with positive appraisals of one’s self and one’s capabilities to cope with stress (Mikulincer and Florian, 1995; Mikulincer, 1998; Mikulincer and Shaver, 2016), with some exceptions (e.g., Sheinbaum et al., 2015). Individuals high in avoidant attachment are thought to engage in inflated self-enhancement when faced with stress (Mikulincer and Florian, 1995; Mikulincer, 1998). However, CSE perceptions specific to the context of recovery from a traumatic event are distinct from self-appraisals in other contexts (e.g., Bandura, 1997; Benight and Bandura, 2004), such as self-efficacy to cope with military training (Mikulincer and Florian, 1995) or generalized self-appraisals (Mikulincer, 1998). Self-efficacy in one area of functioning would not necessarily be expected to have large carry-over effects to self-efficacy in another area of functioning. Specifically, a general sense of mastery in coping independently with other life stressors may not enhance CSE following trauma exposure.

In one prospective study examining pre-traumatic event general self-efficacy (GSE) and the relationship with post-event CSE, it was found that when survivors experienced barriers to mobilizing social support, GSE made minimal impact on post-event CSE (Smith et al., 2017). Thus, the conditions characterizing the social-environmental aspects of the trauma adaptation process moderated the extent to which prior self-efficacy carried over to self-efficacy perceptions specific to trauma recovery. For those high in avoidant attachment, the internal working model of others as untrustworthy or unreliable may act as a self-imposed barrier to mobilizing social supports, diminishing the effectiveness of pre-event self-efficacy perceptions in preventing distress.

Whereas both anxious attachment and avoidant attachment are associated with negative perceptions of perceived social support, they may be distinguished by help-seeking intentions, with avoidantly attached adults less inclined to seek support (Vogel and Wei, 2005). In terms of coping strategies, this avoidant internal working model of others as unhelpful or untrustworthy may manifest itself through distancing coping strategies (Mikulincer et al., 1993; Mikulincer and Florian, 1995). Whereas those high in avoidant attachment may have generally positive self-appraisals, exposure to a traumatic event may overwhelm crucial internal coping resources, leaving one with few other options for coping strategies perceived as viable. Given a sufficient level of distress following trauma and a perceived lack of support, CSE-T would be depleted and PTSD symptoms exacerbated. Future studies may incorporate perceived social support, coping strategies and severity of distress to elucidate the function of CSE-T as an internal coping resource over time.

The interaction between anxious and avoidant attachment was not significantly associated with either CSE-T or PTSD symptoms, suggesting that both insecure attachment orientations have their detrimental effects on self-regulation independent of each other following trauma exposure. A potential factor driving this pattern of findings may be the type of trauma experienced. Specifically, the defenses seen in dismissive individuals may be more effective when faced with a trauma that more directly challenges the attachment system, such as traumatic loss (e.g., Fraley and Bonanno, 2004). Whereas many traumatic events may activate the attachment-based self-regulatory system (Mikulincer and Shaver, 2016) or indirectly challenge the attachment system by straining close relationships (e.g., Taft et al., 2011), other types of traumatic events may be more uniquely relevant to the attachment system, such as interpersonal trauma perpetrated by a loved one or the accidental or violent death of a loved one.

As hypothesized in the present study, some have suggested that an avoidant attachment orientation reduces risk for distress only when accompanied by a low level of anxious attachment (e.g., dismissive attachment; Fraley and Bonanno, 2004; Woodhouse et al., 2015). Those who are prototypically dismissing in their attachment orientation may demonstrate skill in suppressing attachment-related thoughts that lead to distress (e.g., abandonment, rejection, separation; Fraley and Shaver, 1997), which may allow dismissive individuals to maintain appraisals of one’s self as strong and independent (Mikulincer and Shaver, 2016). However, such defenses may be less effective under prolonged stress (Mikulincer and Shaver, 2003) or high cognitive load (Mikulincer et al., 2004). The present sample of survivors were highly trauma-exposed, with high percentages of the sample endorsing exposure items to numerous traumatic events. Further, data collection occurred in the context of an ongoing potentially traumatic event, the SARS-CoV-2 pandemic. One could argue that having either high anxious attachment or high avoidant attachment would demonstrate stronger effects during this level of trauma exposure.

### Limitations

Whereas the present study does benefit from the use of longitudinal data, the use of two timepoints may fall short of expectations for mediation analyses. To establish temporal precedence in a mediational model, it is recommended that one use three separate timepoints (Kline, 2016). Another notable limitation is the representativeness of the present sample. Although prior work has found self-report measures of attachment to be invariant across gender (Gray and Dunlop, 2019) and various ethnic groups (Wei et al., 2004), levels of attachment anxiety and attachment avoidance may vary by key demographic variables. For example, attachment avoidance tends to be greater amongst African Americans and Asian Americans as compared to White Americans (Wei et al., 2004). As the present sample was majority White, Female, and well educated, generalizability of findings to more diverse groups may be limited. As with any research utilizing self-report measures, our findings lack the benefit of originating from clinician-administered interviews and are subject to participants’ biases in reporting.

Finally, the collection of data occurred during the SARS-CoV-2 pandemic. Given the presence of an ongoing threat to health of one’s self and of loved ones, as well as disconnection from some attachment figures (e.g., close friends), our primary variables may have been impacted. It is possible that distress, feelings of isolation, and perceived coping inefficacy may have been exacerbated beyond what would be anticipated following trauma exposure alone. A number of environmental and social factors may impact close relationships during the SARS-CoV-2 pandemic, including (but not limited to) relationship status, quality of relationships, the degree of adherence to social distancing measures, and efforts to maintain contact with friends and family. Stay-at-home orders and social distancing behaviors have been found to be associated with greater depression, anxiety, insomnia, and acute stress in one cross-sectional study of adults in the United States (Marroquin et al., 2020). We did identify one study of the Italian general population on attachment orientation and distress during the pandemic. They found anxious attachment to be a risk factor for greater distress (OR = 1.08), whereas secure and avoidant attachment orientation was protective against distress (OR = 0.89 and 0.92, respectively) following a government mandated lockdown (Moccia et al., 2020).

## Conclusion

Among the sample of survivors exposed to a criterion A trauma in the last year, global attachment plays an important role in explaining variance in PTSD symptoms experienced through CSE appraisals. Appraisals of one’s efficacy to cope with trauma symptoms and demands of recovery (i.e., CSE) represent a mechanism by which adult attachment orientations influence PTSD symptoms six weeks later. Insecure attachment orientations, including anxious and avoidant attachment, were both indirectly associated with PTSD symptoms via trauma specific CSE. The interaction between avoidant and anxious attachment was not significantly associated with either CSE-T or PTSD symptoms. Overall, the present study highlights the role of appraisals in how adult attachment orientations impact self-regulation among trauma survivors. These findings have implications for interventions, suggesting efficacy enhancing support through caregivers and psychotherapy for insecurely attached trauma survivors may be particularly important.

## Data Availability Statement

The raw data supporting the conclusions of this article will be made available by the authors, without undue reservation.

## Ethics Statement

The studies involving human participants were reviewed and approved by Institutional Review Board at the University of Colorado Colorado Springs. The patients/participants provided their written informed consent to participate in this study.

## Author Contributions

MM was responsible for the design and conception of the study, data management, analysis, writing the initial draft, and revising the manuscript. CB was responsible for providing critical revisions to the study design and manuscript. Both authors contributed to the article and approved the submitted version.

## Conflict of Interest

The authors declare that the research was conducted in the absence of any commercial or financial relationships that could be construed as a potential conflict of interest.

## Publisher’s Note

All claims expressed in this article are solely those of the authors and do not necessarily represent those of their affiliated organizations, or those of the publisher, the editors and the reviewers. Any product that may be evaluated in this article, or claim that may be made by its manufacturer, is not guaranteed or endorsed by the publisher.
